# Coniferous Bark as Filler for Polylactic Acid-Based Biocomposites

**DOI:** 10.3390/polym16182669

**Published:** 2024-09-22

**Authors:** Wojciech Jasiński, Radosław Auriga, Seng Hua Lee, Łukasz Adamik, Piotr Borysiuk

**Affiliations:** 1 Institute of Wood Sciences and Furniture, Warsaw University of Life Sciences-SGGW, 159 Nowoursynowska St., 02-776 Warsaw, Poland; radoslaw_auriga@sggw.edu.pl (R.A.); lukasz.adamik@nowystyl.com (Ł.A.); 2 Department of Wood Industry, Faculty of Applied Sciences, Universiti Teknologi MARA, Cawangan Pahang Kampus Jengka, Bandar Tun Razak 26400, Malaysia; leesenghua@uitm.edu.my; 3 Nowy Styl sp. z o.o., ul. Pużaka 49, 38-400 Krosno, Poland

**Keywords:** wood–polymer composites, filler, bark, sawdust

## Abstract

This study explores the possibilities of utilisation of coniferous bark as a filler in wood–polymer composites (WPCs), its impact on properties such as the modulus of rupture (MOR), modulus of elasticity (MOE), thickness swelling (TS) and water absorption (WA) after 2 h and 24 h of immersion in water and the significance of this impact compared to other factors. Six variants of bark–polylactic acid (PLA) WPCs were manufactured, differentiated by their filler content and filler particle size. As a comparison, analogous composites filled with coniferous sawdust were also manufactured. Bark-filled composites were characterised by lower TS and WA after both 2 h and 24 h of immersion, as well as lower water contact angles and surface free energy. The bark filler decreased the composites’ MORs and MOEs, while greater differences were noticed for variants filled with small particles. The type of filler was the second most important factor contributing to variance in this study, with the filler content being the most important one.

## 1. Introduction

Wood–polymer composites (WPCs) provide a cost-effective alternative to both polymer- and wood-based products. While construction is one of the main areas of WPC utilisation, due to their lower thermal stability and material creep, these composites are usually limited to non-load-bearing applications [1]. Other fields in which these composites are widely used include furniture and interior car parts [2]. One of the main advantages of WPCs is the wide spectrum of manufacturing processes that can be used to shape them. Thermoplastic-based composites may be moulded or extruded, as well as machined using equipment dedicated for conventional wood products [3,4].

The physico-mechanical properties of WPCs are usually in the same range as those of a polymer when used as a matrix and wood when used as a filler [5]. Despite this, WPCs should be regarded as neither wood-filled polymers nor polymer-modified wood, but as a completely separate group of materials [6]. WPCs are widely considered to be biocomposites, although most of them are manufactured using petrol-derived polymers, such as polyethylene (PE), polypropylene (PP) and polyvinyl chloride (PVC) [7,8]. Whether WPCs based on non-biodegradable polymers are biocomposites is debatable, but the use of biodegradable polymers (biopolymers), such as polylactic acid (PLA), polyhydroxyalkanoates (PHAs) or thermoplastic starch (TPS), as WPC matrices is also possible [9]. PLA is a plant-derived polymer with properties similar to those of polystyrene (PS) and polyethylene (PE) [10]. It is fully biodegradable, but only under specific conditions, which promotes the long service life of this polymer [11]. PLA is currently regarded as the most extensively researched and used biopolymer [12]. Compared to other commonly used polymers, PLA is characterised by a lower melting point and heat deflection temperature, higher stiffness and lower impact resistance [13].

Wood used in WPCs as a filler is usually a by-product of industrial wood processing in the form of wood shavings, sawdust or wood flour [14]. The choice of wood type and form impacts the physico-mechanical properties of the manufactured composites [15]. Coniferous species are preferable due to their higher slenderness and the simplicity of their anatomical structure [16], while the size of wood particles depends on the WPC’s intended use [17]. Overall, there are no limitations to the utilisation of different wood types as fillers in WPCs [18].

According to Gozdecki et al. [19] and Avci et al. [20], bark used as a filler decreases WPCs’ mechanical properties, such as their modulus of rupture and modulus of elasticity, compared to wood-flour-filled WPCs. Çetin et al. [21] tested composites based on high-density PE and reported that the addition of Turkish pine bark as a filler reduced the tensile and impact strength, while the bending strength and modulus of elasticity improved. Najafi et al. [22] reported that bark decreases the thickness swelling and water absorption of WPCs, in turn improving their moisture resistance compared to traditional WPCs. These correlations were also confirmed by Farasi [23], who tested a WPC filled with beech bark flour. Obuch et al. [24], as part of the Dendromass4Europe project, showed that it is possible to produce WPC composites with a partial 5% replacement of beech flour with crushed poplar bark. While the impact of the bark filler on the aforementioned properties can be confirmed in the literature, the available research lacks the context of other factors impacting WPCs’ performance, such as the filler content or filler particle size. Most of the research also concerns WPCs that are manufactured on the basis of typical petroleum-based thermoplastics (e.g., PE, PP, PVC).

The aim of this study is to evaluate the possibility of manufacturing WPCs based on PLA filled with bark, the impact of the bark filler on selected physico-mechanical properties of WPCs and the significance of that impact compared to the filler content and its particle size.

## 2. Materials and Methods

**Figure 1:**
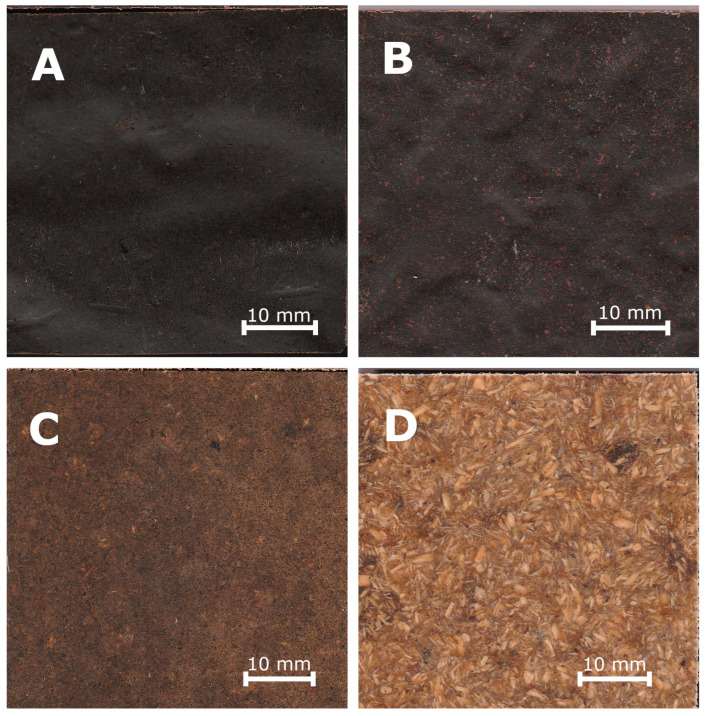
Photos of selected manufactured composites: (**A**) variant XI (60% share of small particles of bark, 40% PLA); (**B**) variant V (60% share of big particles of bark, 40% PLA); (**C**) variant XII (60% share of small particles of sawdust, 40% PLA); (**D**) variant VI (60% share of big particles of sawdust, 40% PLA).

Twelve variants of WPC panels based on polylactic acid (PLA Ingeo™ Biopolymer 2003D, NatureWorks LLC, Minnetonka, MN, USA) were manufactured for this study. The manufactured biocomposites varied in terms of type, size and filler content. Coniferous bark and sawdust, obtained from a sawmill, were used as fillers. The fillers were dried to a moisture content of 5% and then ground and sorted into two groups—particles passing through a 2 mm sieve and remaining on a 0.49 mm sieve (big particles) and particles passing through a 0.49 mm sieve (small particles). Photos of selected manufactured composites are shown in Figure 1. The produced variants are shown in Table 1.

The composites were manufactured in a two-stage process. The first stage consisted of creating a WPC granulate using the Leistritz Extrusionstechnik GmbH (Nürnberg, Germany) extruder and then grinding the composite using a hammer mill. Individual extruder sections were kept at 170–180 °C.

In the second stage, the granulate was used to produce boards sized 300 mm × 300 mm × 2.5 mm by flat pressing in a mould using a single-shelf press. The temperature of the press was set at 200 °C, and the pressure was gradually increased from 0 to a maximum of 1.25 MPa as the materials were plasticizing. The boards were pressed for 6 min, then cooled in the mould for 6 min in a cold press. The manufactured panels were finally conditioned for 7 days at a temperature of 20 ± 2 °C and 65 ± 5% humidity.

For the manufactured panels, the following tests were performed:

- Modulus of rupture (MOR) and modulus of elasticity (MOE) using 3-point flatwise bending tests according to EN 310 [25].
- Density according to EN 323 [26].
- Thickness swelling (TS) and water absorption (WA) after 2 h and 24 h of immersion in water according to EN 317 [27]. Samples were immersed in water with their faces vertical and separated from the edges of the container, as well as each other. The water temperature was maintained at 20 ± 1 °C for the whole duration of the test. After the test time had elapsed, excess water was removed and the samples were weighed and measured.

The tests described above involved ten replicates.

- Wettability (contact angle) was determined by the sessile drop method using a Phoenix 300 (Surface Electro Optics, Suwon City, Korea) contact angle analyser, equipped with microscopic lenses and a digital camera. The contact angles were measured after 20, 40 and 60 s of distilled water droplets touching the surface. Three replicates of these tests were performed.
- Surface free energy was determined by measuring the contact angles of distilled water and diiodomethane after 5 s using the same procedure. The Owens–Wendt [28] method was used to calculate the free surface energy.

Analysis of variance (ANOVA) with a post hoc Tukey test was used to determine the significance of differences between variants, as well as individual factors’ contribution to these differences. Both tests were performed in PQStat v.1.8.7 (PQStat Software, Poznań, Poland) with a confidence level of 95%.

## 3. Results and Discussion

**Figure 2:**
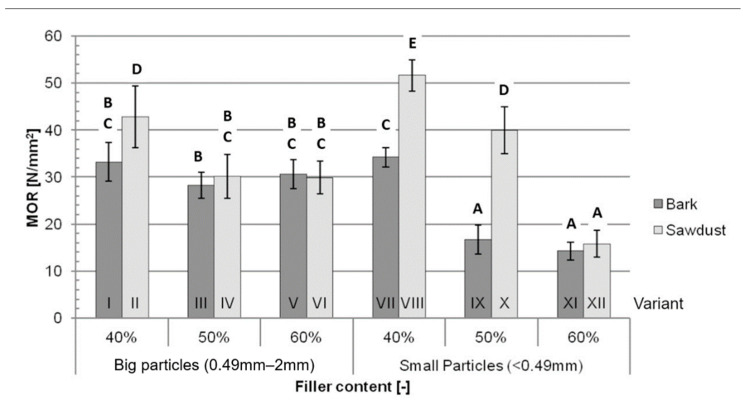
Average MOR for each tested variant: A, B, C, D, E—homogeneous groups, I–XII—composite variant.

Generally, the average MOR (Figure 2) obtained during the study decreased with an increase in the filler content, while being higher for variants containing sawdust than for variants containing bark. These differences were bigger for variants filled with small particles. The average for variant II was 29% higher than the average for variant I, while the average for variant VIII was 51% higher than the average for variant VII. The difference between variant X and XI was 140%, and between variants XII and XI, it equalled 10%, while the differences between variants filled with 50% and 60% big-particle fillers were not statistically significant.

The three most significant factors (Table 2) impacting the MOR were filler content (42.57%), type of filler (15.86%) and an interaction between the particle size and filler content (13.27%). These factors contributed to 71.70% of the variance in this test.

**Figure 3:**
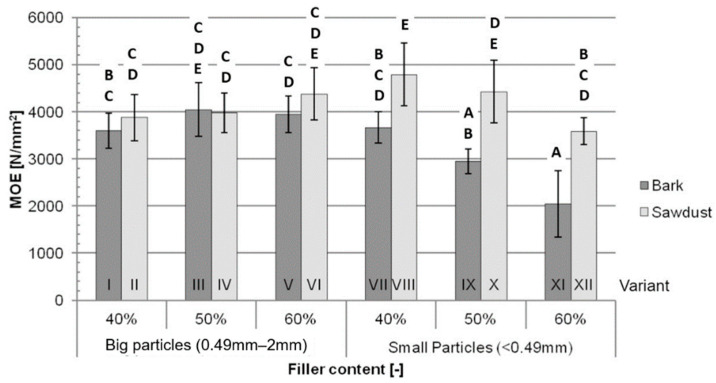
Average MOE for each tested variant: A, B, C, D, E—homogeneous groups, I–XII—composite variant.

Similarly to the averages obtained in the MOR test, bigger differences between the MOE averages (Figure 3) can be seen between variants containing small-particle fillers. The difference between variants with 40% of these fillers is 31%, and between variants filled with 50% and 60% small particles, it is 50% and 76%, respectively. The differences between variants containing big particles are not statistically significant. The average MOE decreases with an increase in small-particle filler content.

The most important factor (Table 2.) impacting the MOE is the type of filler (23.86%), followed by the interactions between the content and the size of filler particles (16.71%) and between the content and type of the filler (12.39%). All three of these factors explain 52.95% of the variance in the MOE test, but each individually impacted the results less than factors that were not considered in this study (Error = 38.29%).

**Figure 4:**
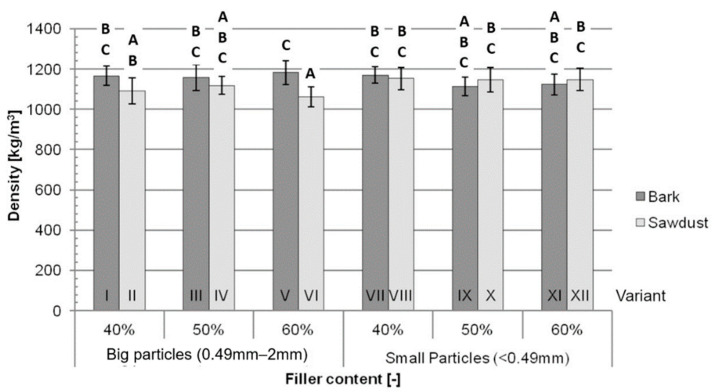
Average density for each tested variant: A, B, C—homogeneous groups, I–XII—composite variant.

The density measured for the tested variants (Figure 4) ranged from 1061 kg/m^3^ for variant VI to 1182 kg/m^3^ for variant V, with an average of 1136 kg/m^3^. Statistically significant differences in density (Figure 3) were only found for the aforementioned variants.

The type of filler (7.24%) and the interactions between the type of filler and size of particles (13.96%) had minor impacts on the measured density, but the most important factors impacting variance in this test (Table 2) were those that were not considered during this study (Error = 69.43%).

According to Jian et al. [1], both the MOR and MOE of WPCs increase with an increase in filler content up to a maximum, which, depending on composite’s formulation, is usually in the range of 40% to 70% filler content. The obtained data suggest that the filler contents used during this study are above the range corresponding to the maximum MOR and MOE for the tested composites. The lower filler content corresponding to the maximum MOR and MOE may be caused by the use of a stiff and brittle matrix, such as PLA, and/or a lack of coupling agent, resulting in a weaker bond between the polymer and fillers. An increase in the tested mechanical properties for the composites filled with small-particle sawdust are in line with the findings of Shah et al. [29] and may be caused by breakage of the fibres being the main failure mechanism. The opposite tendency might be observed for bark-filled composites, which suggests a different failure mechanism. The reason for this difference may be the different polysaccharide composition causing lower adhesion between the filler and matrix. The worse mechanical performance of bark-filled composites might not only be explained by the aforementioned polysaccharide composition, but also by the higher extractives content, more mineral contamination and intrinsically worse mechanical properties of bark itself [30,31]. Compared to traditional high-density fibreboards (HDFs) of similar densities (1050 kg/m^3^ and 1150 kg/m^3^), all the tested variants are characterised by significantly lower MORs and MOEs than those of HDFs [32]. The ANOVA results show that the type of filler itself does not impact the MOR of the tested composites nearly as significantly as the filler content, which might suggest that, by changing the proportions of matrix and filler, a composite with a high bending strength may be manufactured using bark as a filler. These results could be explained by the aforementioned different failure mechanisms. Lower adhesion between the bark and the polymer, coupled with the worse mechanical properties of bark, would suggest that the failure happens due to breakage in the matrix, and a higher content of polymers (lower filler content) would increase the MOR. Additional research, such as microscopic imaging of the broken samples, would be needed to confirm this explanation.

Although the decreases in the tested mechanical properties for variants filled with bark compared to variants filled with sawdust are consistent with the literature, this decrease is not statistically significant for variants filled with 50% and 60% big-particle fillers, which shows that the use of bark as a filler might not impact the mechanical properties of certain WPC formulations in a significant way.

**Figure 5:**
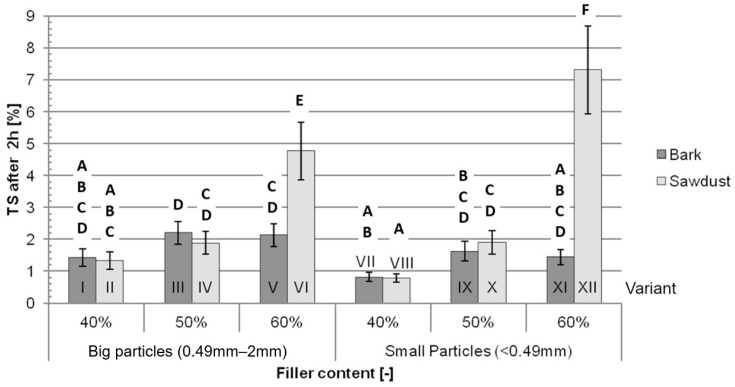
Average thickness swelling after 2 h of immersion in water for each tested variant: A, B, C, D, E, F—homogeneous groups, I–XII—composite variant.

The average TS after 2 h of immersion in water (Figure 5) rises along with the increase in the filler content. The differences between the variants containing bark and containing sawdust are not significant for the composites containing 40% and 50% filler. The average TS after 2 h of immersion for the variants filled with 60% sawdust are higher than the average for variants filled with 60% bark, while these differences are bigger between variants XII and XI, which are filled with small particles (408%), than between variants VI and V, filled with big particles (124%).

**Figure 6:**
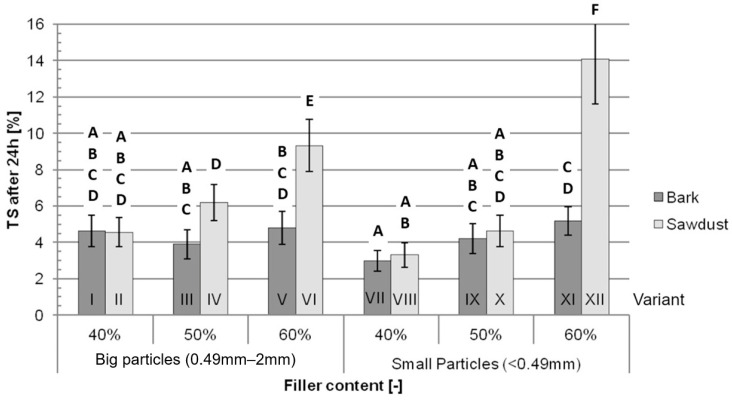
Average thickness swelling after 24 h of immersion in water for each tested variant: A, B, C, D, E, F—homogeneous groups, I–XII—composite variant.

After 24 h of immersion in water, the average TS (Figure 6) for variants IV, VI and XII filled with sawdust is significantly higher than the average TS for variants filled with bark—III, V and XI. The differences between other pairs of variants are not statistically significant. Similarly to the TS after 2 h of immersion, the average TS after 24 h of immersion in water is higher for variants containing more filler.

The most important factors impacting the TS (Table 3) both after 2 h and 24 h of immersion are the filler content (40.05% after 2 h and 37.51% after 24 h), type of filler (13.79% after 2 h and 18.63% after 24 h) and interaction between those two factors (29.15% after 2 h and 20.39% after 24 h). Together, the percentage of the impact of these factors is 82.99% after 2 h of immersion and 76.54% after 24 h of immersion. The particle size itself does not significantly impact the TS in either of those tests.

**Figure 7:**
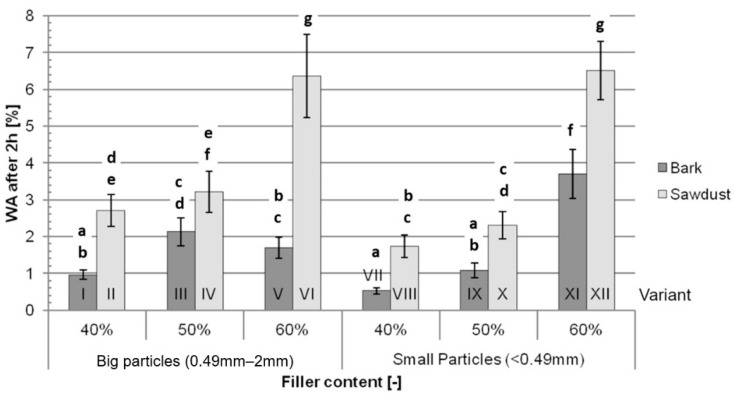
Average water absorption after 2 h of immersion in water for each tested variant: a, b, c, d, e, f, g—homogeneous groups, I–XII—composite variant.

The average WA after 2 h of immersion in water (Figure 7) increases with an increase in filler content while being higher for variants filled with sawdust than for those filled with bark. The differences between pairs of variants differentiated by their filler type range from 51% between variants III and IV up to 277% between variants V and VI.

**Figure 8:**
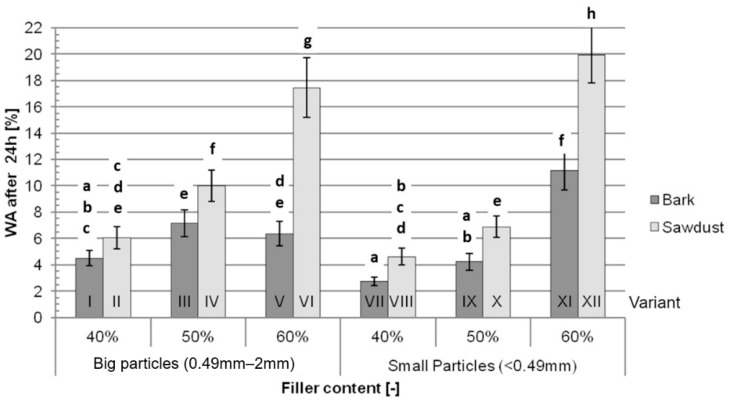
Average water absorption after 24 h of immersion in water for each tested variant: a, b, c, d, e, f, g, h—homogeneous groups, I–XII—composite variant.

After 24 h of immersion in water, the average WA (Figure 8), similarly to the average WA after 2 h, rises with an increase in filler content. Generally, after 24 h, the WA of the variants filled with sawdust is bigger than the WA after 24 h of the variants filled with bark. The difference between variants I and II is not statistically significant, while other differences range from 40% between variants III and IV to 175% between variants V and VI. Those differences are smaller than the differences between variants filled with sawdust and variants filled with bark that were found in the WA after the 2 h test, which suggests that in this study, sawdust-filled WPCs not only absorb more water than analogical bark-filled composites, they also absorb the water faster.

The most important factors impacting WA both after 2 h and after 24 h (Table 3) are the filler content (46.45% after 2 h and 54.56% after 24 h), the type of filler (30.04% after 2 h and 20.66% after 24 h) and the interaction between those two factors (8.80% after 2 h and 12.10% after 24 h). All three of those factors contribute to 85.28% of the variance after 2 h and 87.32% of the variance after 24 h in these tests. Similarly to the TS tests, the size of particles itself impacts WA marginally after 2 h (0.28%) and does not impact WA after 24 h (*p* > 0.05).

The fact that TS and WA increase for variants containing higher filler contents is in line with data available in the literature [33] and is caused by higher amounts of exposed fibres, as well as higher amount of fibres overall. According to Bouafif et al. [34], the particle size of the filler has a minimal impact on water absorption and thickness swelling, which may also be noticed in the data obtained during this study. The lower TS and WA values for variants filled with bark are consistent with literature findings. Despite the fact that lower adhesion between the filler and the matrix, which might be inferred from the results of the mechanical tests, promotes water absorption in WPCs, the hydrophilic properties of lignocellulosic fibres themselves have a bigger impact on TS and WA [20,22]. The differences observed during this study may be explained by the different chemical composition of bark, namely a lower content of hydrophilic cellulose and higher content of possibly hydrophobic extractives. Comparing the results obtained during these tests to the TS and WA values of traditional HDF boards of similar densities (1050 kg/m^3^ and 1150 kg/m^3^), the TS of bark-filled composites, depending on the filler content, range from lower to similar to those of HDFs, while the TS of sawdust-filled composites range from similar to significantly higher than those of HDFs. The WA values of variants with 40% and 50% filler contents and variant 5, filled with 60% big-particle bark, are significantly lower than the WA of HDF. The WA of other variants range from similar to higher than that of HDF [32].

**Figure 9:**
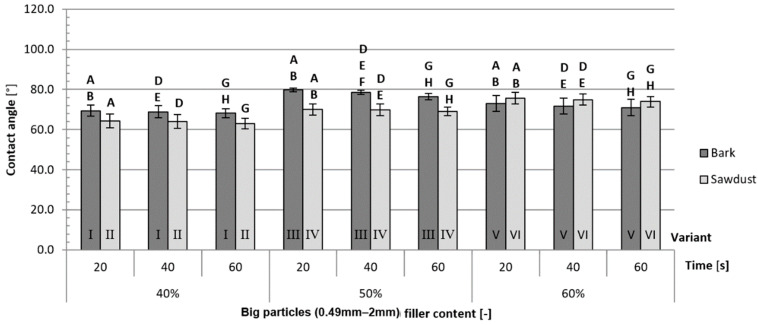
Average contact angles for variants filled with big-particle fillers: A, B, D, E, F, G, H—homogeneous groups, I–XII—composite variant.

**Figure 10:**
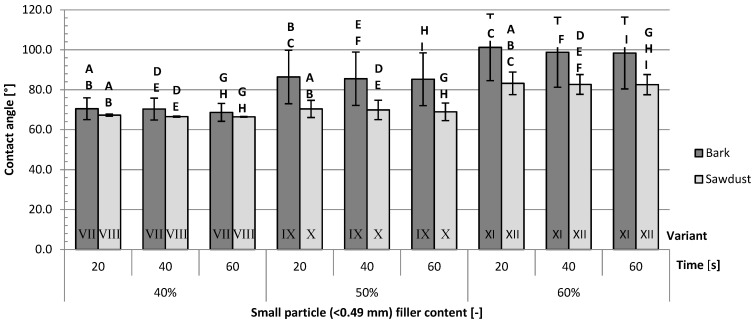
Average contact angles for variants filled with small-particle fillers: A, B, C, D, E, F, G, H, I—homogeneous groups, I–XII—composite variant.

The contact angles of composites filled with big-particle fillers (Figure 9) exhibit not only higher overall values but also bigger differences between variants than the composites filled with small-particle fillers (Figure 10). Apart from variants filled with 60% big-particle fillers, the average contact angles are higher for variants filled with bark than for those filled with sawdust. The differences between the aforementioned variant V and variant VI are not statistically significant. Those findings are true for all tested contact times. The largest differences were measured between variants IX and X (respectively, 19%, 19% and 18% after 20, 40 and 60 s) filled with 50% small-particle fillers. The smallest differences were observed between variants V and VI (respectively, 3%, 3% and 4% after 20, 40 and 40 s), filled with 60% big-particle fillers.

The most important factor impacting the contact angles (Table 4) after either 20, 40 or 60 s is the type of filler. It contributed to 30.56%, 29.35% and 29,66% of the variance, respectively, after 20, 40 and 60 s of contact time. The second most important factor was the filler content for 20 s (12.98%) and 40 s (11.93%) of contact time and the size of the filler particles for 60 s (13.00%) of contact time.

The total surface free energy (Table 5) is higher for the variants filled with sawdust than for the variants filled with bark. The polar component calculated for the variants filled with bark is significantly lower than that of the variants filled with sawdust, with an exception for variants V and VI, filled with 60% large-particle fillers. The disperse component for variants I and VIII filled with bark is higher than that of the analogous variants filled with sawdust, and the inverse is true for all other pairs of variants. The differences in surface free energy are more significant between composites filled with small-particle fillers, which might be explained by the higher specific surface area of smaller particles.

Both the contact angle and surface free energy tests show that bark raises the composite’s hydrophobicity, which positively impacts its water resistance. This may confirm that lower the TS and WA values of the bark-filled composites are a result of the different chemical composition of bark. While the type of filler is not the most important factor in the TS and WA tests, its impact on those results is major, especially under short-term water exposure. The higher impact of the filler content is caused by low PLA WA (below 1% after 24 h of immersion) and insignificant TS [35]. While also considering the ANOVA results for the contact angle tests, the use of bark as a filler should be considered one of the most significant improvements to PLA-based WPC’s water resistance properties.

These results not only show better resistance to water but might also suggest that, for composites used in wet conditions, the impact of a bark filler on the mechanical properties of a tested material might be smaller due to lesser humidity-induced degradation [36].

## 4. Conclusions

The conducted research allows us to conclude that bark can be an alternative to wood sawdust to use as a filler in WPCs. However, the use of bark as a WPC filler causes a decrease in the MOR and MOE of the produced composite compared to the composite with wood sawdust as a filler. The thickness swelling and water absorption of bark-filled WPCs are lower than those of sawdust-filled WPCs. These differences are more significant for composites with higher filler contents. Similarly, the wettability of bark-filled WPCs is lower than that of sawdust-filled WPCs, both in terms of contact angles and surface free energy.

The use of bark as a filler in WPCs improves the moisture resistance of a composite, especially during short-term water exposure. The lower flexural properties of bark-filled composites compared to sawdust-filled ones may be caused by a different failure mechanism under a flexural load, caused by lower adhesion between the filler and the matrix, which could possibly be mitigated by the use of a coupling agent. It should be noted that the use of bark itself impacts the MOR significantly less than the filler content, and in certain applications, the decrease in its flexural properties is not significant.

## Figures and Tables

**Table 1:** Composition of individual variants.

Variant	Share of PLA Matrix	Size of Particles	Kind of Particles
I	60%	(0.49–2 mm)	Bark
II			Sawdust
III	50%		Bark
IV			Sawdust
V	40%		Bark
VI			Sawdust
VII	60%	(<0.49 mm)	Bark
VIII			Sawdust
IX	50%		Bark
X			Sawdust
XI	40%		Bark
XII			Sawdust

**Table 2:** ANOVA for selected factors affecting MOR, MOE and density of tested composites.

Factor	MOR		MOE		Density	
	*p*	P	*p*	P	*p*	P
a	0.000	42.57%	0.009	3.72%	0.361	1.32%
b	0.000	15.86%	0.000	23.86%	0.001	7.24%
c	0.000	2.58%	0.002	3.72%	0.204	1.05%
a × b	0.000	6.40%	0.510	0.51%	0.122	2.75%
a × c	0.000	13.27%	0.000	16.71%	0.258	1.77%
b × c	0.000	5.67%	0.000	12.39%	0.000	13.96%
a × b × c	0.000	3.06%	0.340	0.82%	0.361	1.32%
Error		10.58%		38.29%		69.43%

a—filler content, b—type of filler, c—size of particles, *p*—probability of error, P—percentage of contribution.

**Table 3:** ANOVA for selected factors affecting TS and WA after 2 h and 24 h of immersion in water.

Factor	TS after 2 h		TS after 24 h		WA after 2 h		WA after 24 h	
	*p*	P	*p*	P	*p*	P	*p*	P
a	0.000	40.05%	0.000	37.51%	0.000	46.45%	0.000	54.56%
b	0.000	13.79%	0.000	18.63%	0.000	30.04%	0.000	20.66%
c	0.835	0.00%	0.430	0.06%	0.038	0.28%	0.144	0.10%
a × b	0.000	29.15%	0.000	20.39%	0.000	8.80%	0.000	12.10%
a × c	0.000	3.04%	0.000	7.54%	0.000	5.48%	0.000	7.38%
b × c	0.000	2.93%	0.020	0.58%	0.000	0.94%	0.101	0.12%
a × b × c	0.000	3.43%	0.000	4.13%	0.000	1.15%	0.041	0.29%
Error		7.60%		11.15%		6.87%		4.80%

a—filler content, b—type of filler, c—size of particles, *p*—probability of error, P—percentage of contribution.

**Table 4:** ANOVA for selected factors affecting contact angles after 20, 40 and 60 s.

Factor	Contact Angle after 20 s		Contact Angle after 40 s		Contact Angle after 60 s	
	*p*	P	*p*	P	*p*	P
a	0.002	12.98%	0.004	11.93%	0.005	10.68%
b	0.000	30.56%	0.000	29.35%	0.000	29.66%
c	0.003	11.55%	0.004	11.62%	0.002	13.00%
a × b	0.327	2.45%	0.404	2.20%	0.378	2.28%
a × c	0.086	3.36%	0.093	3.57%	0.093	3.45%
b × c	0.019	9.80%	0.028	9.76%	0.022	10.05%
a × b × c	0.159	4.16%	0.236	3.58%	0.197	3.91%
Error		25.14%		27.99%		26.96%

a—filler content, b—type of filler, c—size of particles, *p*—probability of error, P—percentage of contribution.

**Table 5:** Surface free energy for each tested variant.

Filler Content	Variant	Surface Free Energy [mJ × m^−2^]		
		γ^tot^	γ^D^	γ^P^
40% L	I	51.68	45.60	6.07
	II	52.23	43.24	8.99
50% L	III	44.33	41.18	3.15
	IV	52.59	47.45	5.14
60% L	V	47.36	42.17	5.20
	VI	48.75	45.18	3.57
40% S	VII	49.40	44.31	5.10
	VIII	49.50	41.84	7.66
50% S	IX	38.29	36.53	1.77
	X	47.22	40.55	6.67
60% S	XI	37.76	37.75	0.00
	XII	50.24	49.17	1.07

L—large particles (0.49–2 mm), S—small particles (<0.49 mm), dark grey—bark, light grey—sawdust.

## Data Availability

The original contributions presented in the study are included in the article, further inquiries can be directed to the corresponding author.
